# Survival prognostic factors in patients undergoing cytoreductive surgery and hyperthermic intraperitoneal chemotherapy treatment: analysis from a single oncological center

**DOI:** 10.1186/s12957-016-0856-y

**Published:** 2016-03-31

**Authors:** L. Graziosi, E. Marino, V. De Angelis, A. Rebonato, A. Donini

**Affiliations:** Department of General and Emergency Surgery, Santa Maria della Misericordia Hospital, University of Perugia, Via Dottori, 06132 Perugia, Italy; Department of Clinical Oncology, Santa Maria della Misericordia Hospital, University of Perugia, Perugia, Italy; Department of Radiology, Santa Maria della Misericordia Hospital, University of Perugia, Perugia, Italy

**Keywords:** Peritoneal carcinomatosis, Gastrointestinal malignances, Intraperitoneal chemotherapy

## Abstract

**Background:**

The aim of our study is to analyze survival, treatment-related morbidity, and safety in our experience of cytoreductive surgery and hyperthermic intraperitoneal chemotherapy.

**Methods:**

Sixty-four patients were treated. Survival curves were calculated according to the Kaplan-Meier method. Univariate and multivariate analyses were done, and Cox’s proportional hazard model was used to identify significant factors.

**Results:**

Global 5-year overall survival was 55 %. Overall survival was also evaluated according to neutrophils to lymphocytes ratio and neutrophils to platelets ratio. Overall survival according to pre-operative serum albumin level shows a difference in the two groups (*P* < 0.05). We observed minor or no adverse events in 53 cases (89.8 %), while 3 patients (5.1 %) showed a grade III–IV complication and 3 post-operative deaths (5.1 %). Post-operative complication also influenced overall survival; patients in whom a minor complication occurred had a 3-year overall survival (OS) of 62 % vs. a 3-year OS of 28 % in patients who underwent a major complication (*P* < 0.1).

**Conclusions:**

Hyperthermic intraperitoneal chemotherapy (HIPEC) could be a valid and feasible option for selected patients affected by gastrointestinal malignancies’ peritoneal carcinomatosis.

Pre-operative parameters could be evaluated to choose patient who could benefit from cytoreductive surgery and hyperthermic intraperitoneal chemotherapy.

## Background

Peritoneal carcinomatosis (PC) has been considered as a rapidly lethal disease and therefore mainly managed by palliative options and conservative care.

Gastroenteric and ovarian cancers behave as the main actors in the development of PC, which leads the patients to a very poor prognosis [1–3].

The treatment of PC is an expanding area in which a multimodality therapy approach has been shown to significantly increase overall survival.

Recently, the clinical interest on this clinical condition has increased for the encouraging results reported combining cytoreductive surgery (CRS) and hyperthermic intraperitoneal chemotherapy (HIPEC) [4–6].

Surgical cytoreduction aims to remove all the peritoneal visible disease, whereas HIPEC treatment attempts to eliminate the occult carcinomatosis for the presence of free cancer cells in the peritoneal cavity.

This complex approach could include an extensive peritonectomy, multiple organs resection, intraperitoneal chemotherapy, and hyperthermia. Not surprisingly, many trials reported a high morbidity comparable to that of major surgery (Whipple procedure, gastrectomy with an extensive lymphadenectomy) [7–9].

It has become crucial to understand whether we can consider prognostic factors than can predict patients’ outcomes and post-surgical complications.

Although it is recognized that the development of cancer has a genetic basis, there is increasing evidence that the host inflammatory response plays an important role in the development and progression of cancer. Inflammatory biomarkers, such as neutrophil to lymphocyte ratio (N/L), neutrophil to platelet ratio (N/P), and serum albumin level, hold great promise for improving the predictive ability of existing prognostic tools in cancer patients [10]. N/L ratio seems to be related to the prognosis of various types of cancer. In particular, a high N/L ratio has been suggested to be associated with poor outcome [11].

Moreover, in advanced tumors, a high pre-operative C-reactive protein (CRP) level and/or high platelet count were frequently observed and were associated with poor patient prognosis [12].

The aim of our study is to analyze overall survival (OS), treatment-related morbidity, and safety in our initial experience considering various prognostic factors and their survival impact in patients undergoing CRS and HIPEC.

## Methods

Sixty-four consecutive patients with PC from different primitive tumors have been treated in our center from September 2006 to September 2014. A prospective collected database has been created and data were retrospectively analyzed.

Eligibility requirements included the following: diagnosis of peritoneal surface malignancies (PSM) made or confirmed in our Pathology Department, age 75 years or younger, performance status 2 or less according to Eastern Cooperative Oncology Group (ECOG), no significant comorbidities, no extra-abdominal or hepatic metastases in number superior to 3, and pre-operative computer tomographic (CT) scan showing peritoneal disease amenable to potentially complete surgical cytoreduction.

Average age was 59.6 ± 10.8 years old.

Median follow-up was 26.5 months (range 4–120 months).

At the beginning of the surgical procedure, the abdomen was explored and Peritoneal Cancer Index (PCI) was calculated by dividing the abdomen region into 13 quadrants and assigning a lesion size score according to Sugarbacker carcinomatosis index [13]. Patients that underwent adjuvant HIPEC treatment (gastric cancer patients at high risk to develop PC with serosal involvement or positive peritoneal cytology) were considered with a PCI equal to zero.

Median PCI was 2 (range 0–19).

Patients were stratified into three different groups according to PCI evaluation (0–6; 7–10; > 10).

Five patients (7.8 %) underwent cytoreductive surgery without HIPEC treatment and therefore have been excluded from the current study.

Three patients (4.7 %) did a laparoscopic palliative HIPEC treatment for malignant ascites from breast cancer (1 case) and pleuric mesothelioma (2 cases). Of the other 56 cases (87.5 %), in 44 cases (22 colon cancer, 9 ovaric cancer, 9 gastric cancer, 2 jejunal carcinoma, 1 peritoneal mesothelioma, 1 endometrium), HIPEC treatment was used in a therapeutic setting (PCI > 0).

In 12 (18.7%) patients, HIPEC was performed as adjuvant treatment for locally advanced gastric cancer.

In two patients (3.1%), an iterative procedure was performed due to peritoneal recurrences from ovarian cancer.

CRS was performed in all the patients, with the intent to remove all the macroscopic disease. Anterior abdominal peritonectomy, right and left diaphragmatic peritonectomy, right and left parietocolic peritonectomy, greater omentectomy, lesser omentectomy, and pelvic peritonectomy were performed during the surgical procedures together with the resection of the abdominal organs compromised by the tumor.

Small and scattered localizations on visceral surfaces were resected by local excision or electrocoagulation. All anastomoses were performed before the beginning of the intraperitoneal perfusion. Protective ostomies were performed only in high-risk patients after HIPEC, to prevent perfusate leak from ostomy tracts through the abdominal wall.

HIPEC was performed according to the closed-abdomen technique, at temperature of 42.5 °C, with cisplatin (25 mg/m2/L) plus doxorubicin (15 mg/L) for 90 min or cisplatin (25 mg/m2/L) plus mitomycin-C (3.3 mg/m2/L) for 60 min or oxaliplatin (360 mg/L/m2) for 30 min at the temperature of 43 °C. Perfusate volume was 4 to 6 L; average flow was 700 mL/min. The Performer LRT (RAND, Medolla (MO), Italy) extracorporeal circulation device was used.

The completeness of cytoreduction (CCR) [14] was classified at the end of the surgical phase, as macroscopically complete (CCR-0); nearly complete: residual disease 2.5 mm or less in any region (CCR-1); or suboptimal: residual disease more than 2.5 mm (CCR-2).

All the patients were admitted to the intensive care unit (ICU) after the surgical procedure for at least 24 h.

Venous blood sampling was taken before surgery and collected in ethylenediaminetetraacetic acid (EDTA) containing tube. The normal range of white blood cells (WBC) count was from 4000 to 10,800 cells/mm^3^.

Baseline N/L ratio and N/P ratio were calculated as neutrophil absolute number count divided by lymphocyte or platelets absolute number count.

Median value of N/L ratio (2.12), N/P (0.27) ratio, and serum albumin (3.8 g/dL) were used to dichotomize patients in two homogenous groups.

A record of any post-operative complications was made according to the National Institutes of Health morbidity and mortality grading system [15].

Adverse events were divided into five categories, as shown according to Clavien-Dindo classification [16].

Morbidity-mortality-related procedure was considered within 60 days from the surgical procedure.

OS was calculated according to the Kaplan-Meier method from the initial date of treatment to the occurrence of the event or to the date of the last follow-up (follow-up in our center is performed for 10 years from the surgical procedure); differences were determined using a log-rank test.

Univariate and multivariate analyses were done, and Cox’s proportional hazard model was used to identify significant factors related with prognosis and complications.

A *P* < 0.05 was regarded as statistically significant.

Research carried out is in compliance with the Helsinki Declaration.

Graph Pad Prism version 5.0 and SAS statistical softwares were used to generate these analyses.

## Results

Between September 2006 and September 2014, 59 consecutive combined procedures were performed.

Clinical and pathological features and some surgical details of the patients are shown in Table 1.

**Table 1:** Clinical pathological characteristics of our patients' series

Variables	No. of patients	Percentage
Median age	59.6 ± 10.58 years old	
M/F	26/38	
CRS without HIPEC	5	7.8
CRS + HIPEC	59	92.2
Primary tumor:		
• Colon	22	34.4
• Gastric	21	32.8
• Ovary	9	14.1
• Other tumor with malignant ascites	3	4.7
• Other	4	6.2
Performance Karnovsky Status (PKS) > 90%	58	90.6
Median PCI	2	
CC:		
• 0	50	89.3
• 1	4	7.1
• 2	2	3.6
Neoadjuvant chemotherapy	15	25.4
Adjutant chemotherapy	55	93.2
Adjutant HIPEC	12	20.3
Palliative HIPEC	3	5.1
Intraoperative drugs:		
• Cysp. + Myto	34	57.6
• Oxaliplatin	14	23.7
• Doxo	4	6.8
• Other	7	11.9

**Fig. 1:**
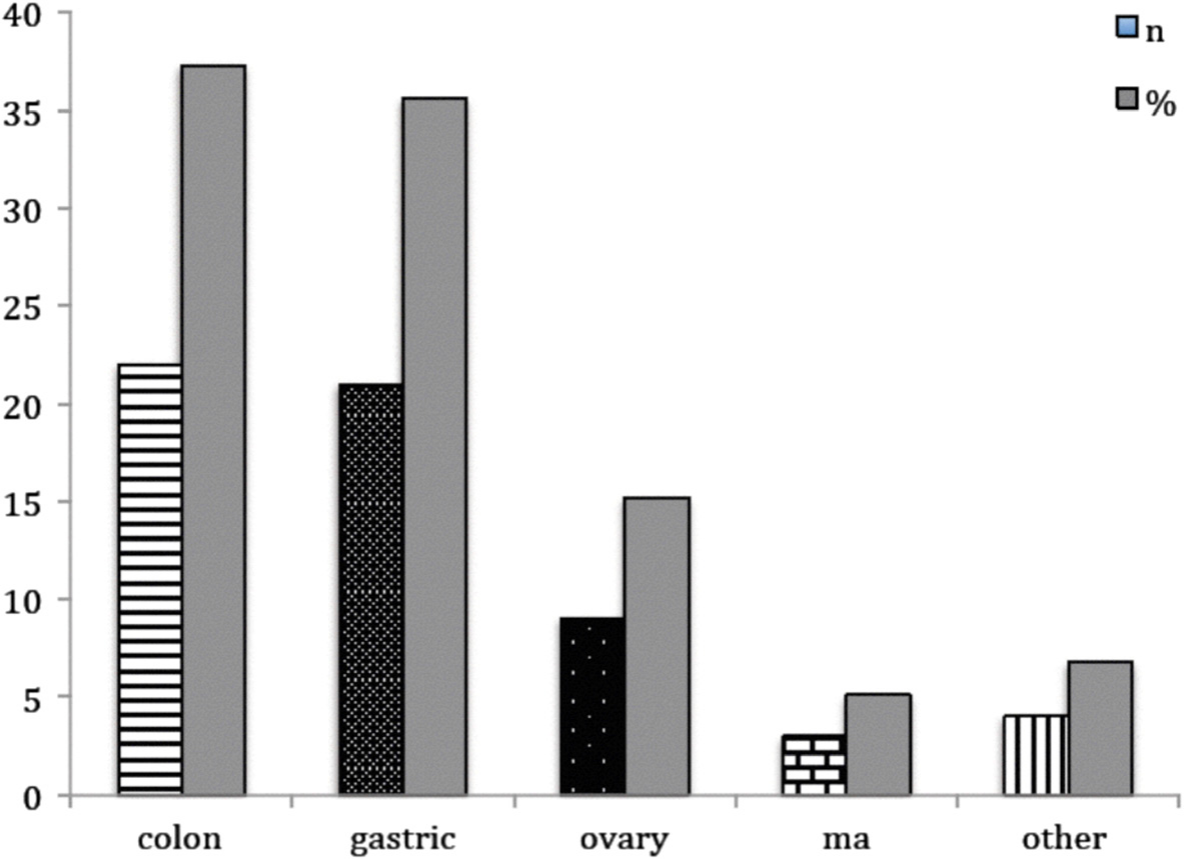
Primary tumor distribution in our series (absolute number and percentage)

We treated 22 PC originating from colon cancer, 21 PC from gastric cancer, nine PC from ovarian cancer, two PC from small intestine, one from peritoneal mesothelioma, and one from the uterus (Fig. 1).

**Fig. 2:**
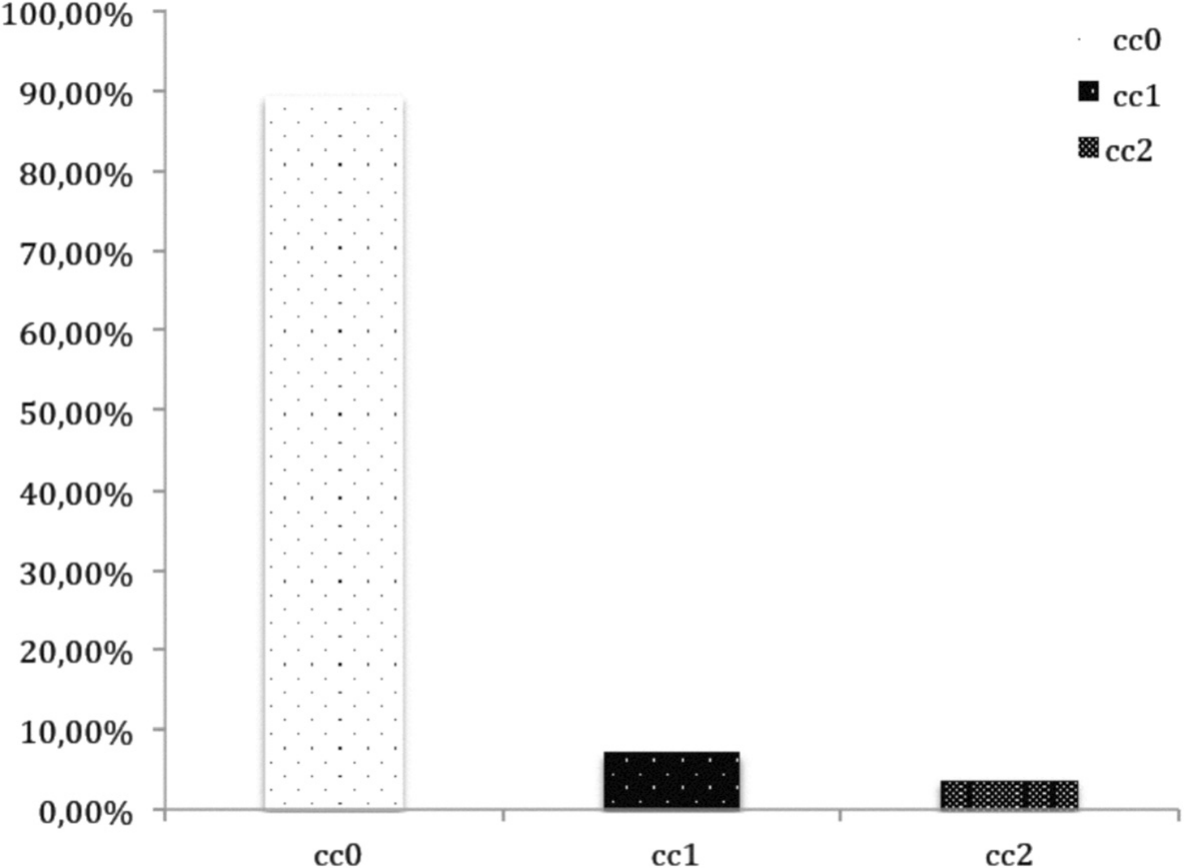
Completeness of cytoreduction distribution in our series (CC score)

Excluding three patients with malignant ascites who underwent palliative laparoscopic HIPEC treatment, CC0 was achieved in 50 patients (89.3 %), CC1 in 4 patients (7.1 %), and CC2 in 2 patients (3.6 %) (Fig. 2).

HIPEC was performed in 34 patients with cysplatin plus mitomycin, in 14 patients with oxaliplatin, and in four with intraperitoneal infusion of doxorubicin.

**Fig. 3:**
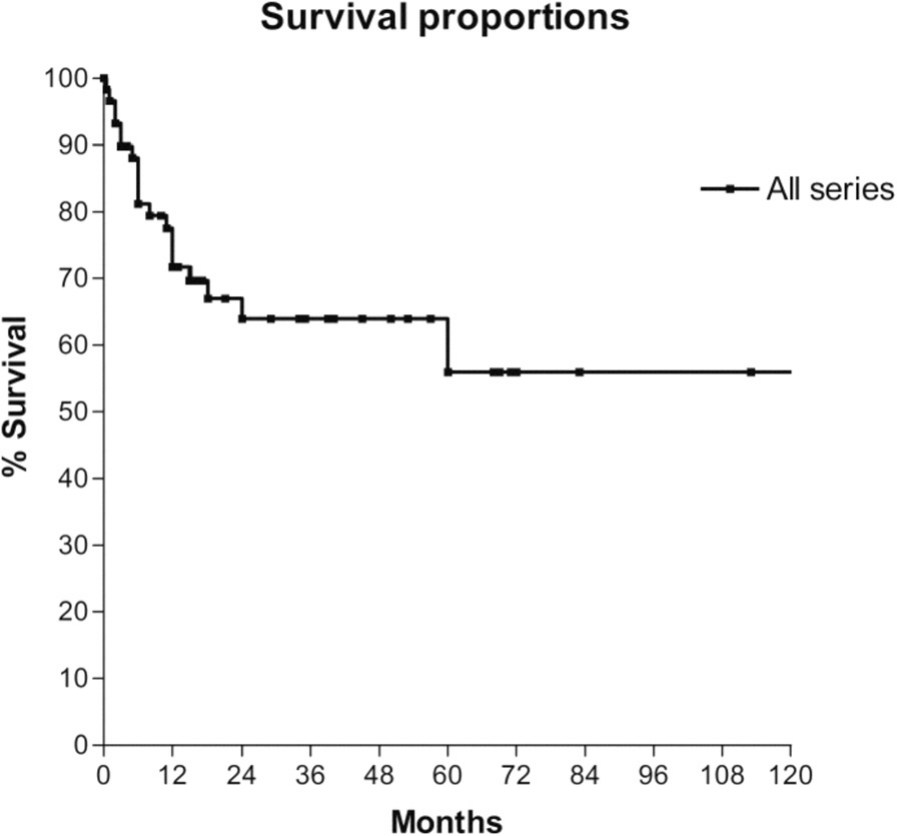
Overall survival (OS) in our series; 5-year OS, 55 %

In our series, we analyzed a global 5-year OS (Fig. 3) that was 55 %.

Then, we evaluated OS according to different variables: PCI, CC score, primitive tumor’s location, pre-operative serum albumin value, pre-operative N/L ratio, pre-operative N/P ratio, adjuvant chemotherapy,and post-surgical complications.

**Fig. 4:**
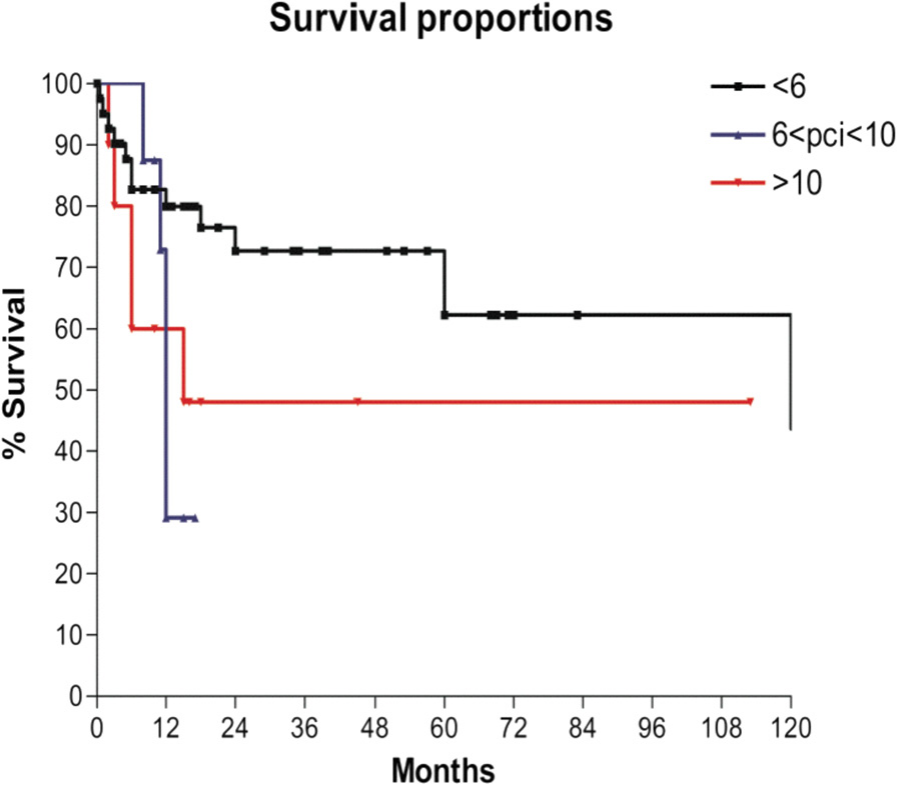
Overall survival according to Peritoneal Cancer Index

According to PCI, patients were divided into three subgroups as previously described and OS show a statistically significant difference (*P* < 0.05) (Fig. 4).

**Fig. 5:**
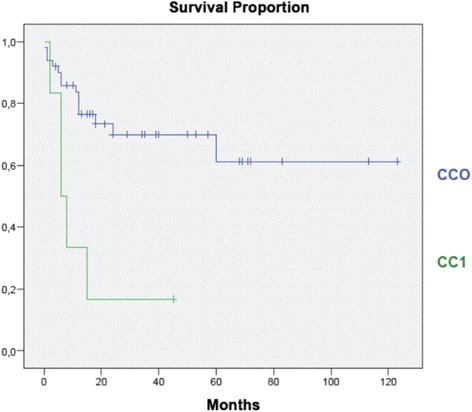
Overall survival according to completeness of cytoreduction (*P* < 0.05)

According to primitive tumor site, OS was evaluated and no statistically significant differences were found, whereas there was a statistically significant differences (*P* < 0.05) between patients who underwent a complete cytoreduction vs. incomplete (Fig. 5).

OS was also evaluated according to N/L and N/P ratio with no statistically significant differences (*P* > 0.05) in the two groups.

**Fig. 6:**
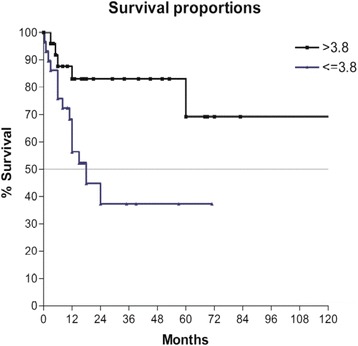
Overall survival according to serum albumin level; *P* < 0.05

However, OS according to pre-operative serum albumin level (Fig. 6) shows a difference in the two groups with a 5-year OS in the higher group of 70 % vs. a 5-year OS of 38 % in the lower group (*P* < 0.05).

We observed minor or no adverse events in 53 cases (89.8 %), while 3 patients (5.1 %) showed a grade III–IV complication.

**Fig. 7:**
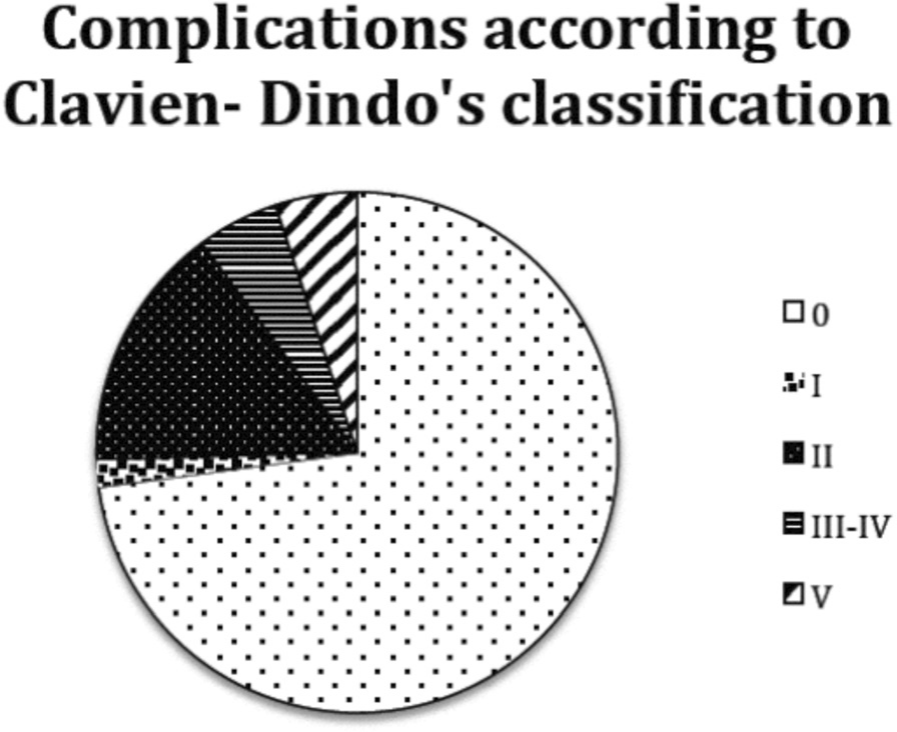
Clavien-Dindo’s complications distribution

There were three post-operative deaths (5.1 %): one due to a necrotic hemorrhagic pancreatitis in patients with an anastomotic leakage and two due to acute respiratory distress syndrome and respiratory failure (Fig. 7).

**Fig. 8:**
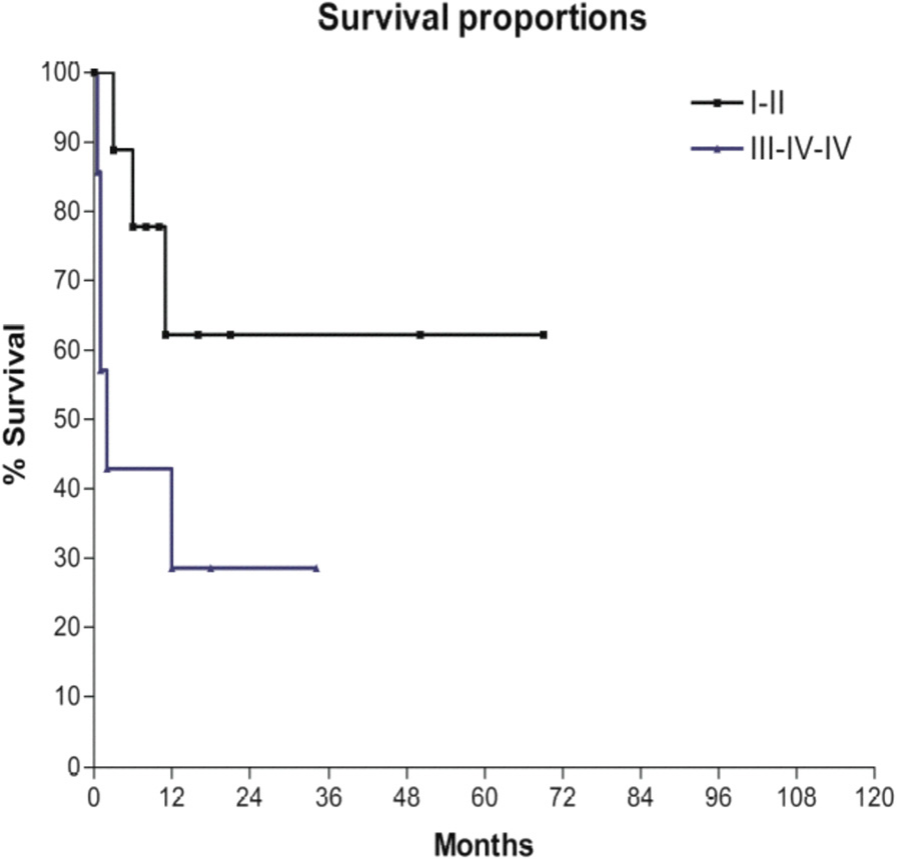
Overall survival according to complications rate *P* < 0.1

Post-operative complication also influenced the OS as shown in Fig. 8; patients in whom a minor complication (grades I and II) occurred had a 3-year OS of 62 % vs. a 3-year OS of 28 % in patients who underwent a major complication (*P* < 0.1).

Performing a univariate analysis using log-rank test, the following factors were correlated with poorer overall survival: serum albumin level (*P* = 0.009); CC score (*P* 0.002); and complication rate (*P* 0.015). Serum albumin level was still significant in the final multivariate model (HR 5.2; 95 % CI 1.5–18.1; *P* = 0.009.) as well as CC score (HR 5.9; 9 5% CI 1.8–18.9; *P* 0.003) and complication rate (HR 5.9; 95 % CI 2.0–17.5; *P* = 0.001).

## Discussion

Peritoneal dissemination is a form of cancer progression, which can frequently affect patients with gastrointestinal and ovarian cancers having a median survival not exceeding 6 months.

For this reason, a handful of centers have pursued aggressive CRS to resect macroscopic disease as much as possible, combining it with HIPEC to treat any residual occult disease.

Nowadays, complete CRS plus HIPEC became the new standard of treatment for peritoneal carcinomatosis giving promising results with an acceptable morbidity.

However, continuous and meticulous analyses are needed to enable further improvements to this technique.

As long as the aim of cancer surgery is to cure, then patient selection remains uncontroversial. Yet, like all other treatments, CRS and HIPEC must achieve the optimum risk benefit balance based on data reported in literature.

In this study, we want to report our HIPEC patient results in terms of overall survival, disease free survival, and post-operative complications and we correlated them to various prognostic factors.

Our aim is to better identify patients who could really benefit of this aggressive treatment with an acceptable morbidity.

Actually, different scoring systems try to select the “right patient” to address to this procedure.

Nowadays, there are a number of pre-operative prognostic models published for PC as the peritoneal surface disease severity score (PSDS) [17], laparoscopic staging [18], or prognostic score [19].

These methods are based primarily on the extent of PC in the abdominal cavity.

In contrast, analyses based on blood samples could provide a simple scoring system.

The colon rectal peritoneal score (COREP) is based mainly on serum tumor markers and their change between referral and surgery. The COREP score reflects the tumor biology and can support radiology to improve the patient selection process before surgery.

There are, however, other prognostic scores, notably the PSDS and the prognostic score (PS), appeared because the pre-operative radiologic evaluation is not able to exclude patients to surgery only on the basis of PC extension [20].

Radiologic examinations could individualize metastatic patients with lung or liver metastases, retroperitoneal lymphonodes that render the patient surgical ineligible.

These scores are more dependent on the extent of the PC to predict survival.

Cashin [21] in his study compared in a univariate and multivariate analyses these three prognostic scores. Although the COREP score was designed to reflect a more tumor biology perspective rather than tumor burden, it still correlated to the PCI. This study demonstrates that the COREP score can identify patients who may quickly experience systemic recurrence, seeming superior to the PSDS, and the PS in identifying patients who will not benefit from CRS and HIPEC.

Recently, Adachi [22] correlated the modified Glasgow Prognostic Score (mGPS) to the 3-month mortality rate in patients who undergo surgical intervention with systemic chemotherapy for synchronous PC from CRC.

The mGPS could aid surgeons in choosing the appropriate treatment strategy and the best care for patients. In fact, according to these study results, mGPS was the only independent risk factor of post-operative mortality.

In our survival analysis, we considered patient-related parameters (pre-operative serum albumin level; pre-operative serum neutrophils to lymphocytes and platelets to lymphocytes ratio) and tumor-related factors (PCI; primary tumor site, CC score).

In addition, we correlated OS to post-surgical complications occurrence.

Pre-operative serum albumin level is strongly correlated to patient survival.

Both in our univariate and multivariate analysis albumin is strongly correlated to patient prognosis. Albumin has been described as a negative inflammatory marker in various studies.

Cancer is generally associated with an inflammatory status of the patients; as a matter of fact, cancer growth and its invasion induce local tissue damages, local homeostasis alteration, and finally a systemic acute-phase response. The major role of the acute-phase response is to remove the cause from the body and to restore the initial status; however, this response persists in case of cancer, and it contributes to the development of the pathology of disease, as cancers are “wounds that do not heal” [23].

Alternatively, cancers continue to progress in a non-self-limiting manner, while inducing their stroma, essential to growth, by activating the host’s wound healing response. Thus, this systemic inflammatory response (SIR) associated with cancer must be ongoing and persistent. SIR is associated with an induced liver production of acute-phase proteins as protein C-reactive; IL-6; and IL-1.

These factors, on the other hand, cause a negative drive to albumin liver synthesis.

Mc Milla et al. [24] also found that a low albumin level was associated with an elevated phase acute proteins level, and they concluded that this was likely a secondary event resulting from ongoing cancer-associated inflammation, not simply from the nutritional decline associated with food intake disturbance.

Thus, both the increasing of C-reactive Protein (CRP) and interleukins and albumin decreasing should be correlated to the advanced tumor stage and poor prognosis.

In advanced tumors, frequently a high pre-operative C-reactive protein level is accompanied by high platelet count associating a poor patient prognosis [25]. The pre-operative neutrophil-lymphocyte ratio (NLR) also reflects patients’ inflammation status, clinical stage, and patients’ survival in colon cancer, lung cancer, and liver cancer [26–31].

Increased numbers of neutrophils and/or decreased numbers of lymphocytes may suppress lymphokine-activated killer cells, thereby increasing the propensity to metastasis.

Our patients’ survival is not statistically correlated to NLR or PLR probably due to the fact that patients underwent chemotherapy before HIPEC and CRS procedure altering their immune status and probably also due to their advanced metastatic disease.

We have previously demonstrated [32] that elevated pre-operative N/L ratio predicts poor overall survival following resection in our gastric cancer patients.

Thus this ratio may be utilized as a simple, reliable prognostic factor for risk stratification but only for patients at an early stage of disease being T and N stages more statistically significant in advanced stage of disease.

As demonstrated in other studies [33–35], PCI is obviously strongly correlated to overall survival. Post-surgical complications affect long-term outcomes; in fact patients, showing major morbidity, have a poorer prognosis compared to patients who did not. Previously Baratti et al. [36] analyzed the clinical impact of treatment related morbidity on survival in patients with colon cancer treated with HIPEC and CRS.

At multivariate analysis, major morbidity correlated to worse overall disease-specific survival independently from PCI value (11.7 vs. 58.8% of patients who did not show major post-surgical complications) comparable to our results.

The mechanisms by which operative complications impact oncologic outcomes remain controversial. Probably, we can explain this correlation according to the following hypothesis: (1) there are some biological factors that predispose patients to both complications and poor long-term survival, (2) operative morbidity directly affects oncologic post-surgical outcome, and (3) surgeon’s experience and learning curve affect directly both early and late post-surgical results.

Regarding the first point, surgery is more technically demanding in patients with advanced-stage of disease, resulting in a higher risk of post-operative complications occurrence.

On the other side, complications may worsen general conditions and they impede subsequent adjuvant therapies or treatment for recurrence that is need in advanced disease.

Furthermore, there is evidence that cell-mediated immunity and natural killer cell function are suppressed by surgical stress. Because the immune system has been suggested to play a role in controlling microscopic residual disease following surgical resection, complications might aggravate the negative impact on immunity and facilitate tumor progression.

We think that prevention of major complication is fundamental and this can be done by doing a careful patient selection and improving surgeon learning curve.

In our study, overall grade III and IV morbidity was very low and mortality was limited to few cases. It could be explained with the low median PCI, the correct patient selection being certainly the procedure’s “Achilles heel,” and with the advanced surgical experience of the center.

## Conclusions

Our study is limited by the small size of patients and by the fact that different primary tumor were analyzed giving heterogeneous results.

However, it seems that CRS and HIPEC could be a valid and feasible option for locally advanced gastrointestinal malignancies reporting good survivals. Pre-operative parameters could be evaluated to rightly choose patient who could really benefit from HIPEC and CRS with an acceptable post-surgical morbidity.
